# Supplementation Effect of a Combination of Olive (*Olea europea* L.) Leaf and Fruit Extracts in the Clinical Management of Hypertension and Metabolic Syndrome

**DOI:** 10.3390/antiox9090872

**Published:** 2020-09-15

**Authors:** Michel P. Hermans, Philippe Lempereur, Jean-Paul Salembier, Nathalie Maes, Adelin Albert, Olivia Jansen, Joël Pincemail

**Affiliations:** 1 Service d’Endocrinologie et de Nutrition and Pôle Endocrinologie, Diabète et Nutrition (EDIN), Institut de Recherche expérimentale et clinique, UCLouvain, 1200 Brussels, Belgium; michel.hermans@uclouvain.be; 2 Service de Cardiologie, Centre Hospitalier Bois de l’Abbaye, 4100 Seraing, Belgium; philippe.lempereur@skynet.be; 3 Service de Cardiologie, CHU UCL Namur - site Sainte-Elisabeth, 5000 Namur, Belgium; jeanpaulsalembier@yahoo.fr; 4 Biostatistics and Medico-economic Information Department, University Hospital of Liège, 4000 Liège, Belgium; nmaes@chuliege.be (N.M.); aalbert@uliege.be (A.A.); 5 Laboratoire de Pharmacognosie, Centre Interdisciplinaire de Recherche sur le Médicament (CIRM), Université de Liège, 4000 Liège, Belgium; ojansen@uliege.be; 6 Department of Cardiovascular Surgery, CREDEC and Platform Nutrition Antioxydante et Santé, CHU and University of Liège, Sart Tilman, 4000 Liège, Belgium

**Keywords:** *Olea europea* L., olive extracts, oleuropein, hydroxytyrosol, hypertension, metabolic syndrome

## Abstract

Background: The role of herbal products in the prevention of cardiovascular disease requires supporting evidence. This open pilot study assessed the effect of 2-month supplementation of a combination of olive leaf and fruit extracts (Tensiofytol^®^, Tilman SA, Baillonville, Belgium) in the clinical management of hypertension and metabolic syndrome (MetS). Methods: A total of 663 (pre)-hypertensive patients were enrolled by general practitioners and supplemented for two months with Tensiofytol^®^, two capsules per day (100 mg/d of oleuropein and 20 mg/d of hydroxytyrosol). Systolic and diastolic blood pressures (SBP/DBP) were measured before and after treatment. Markers of MetS, high-density lipoprotein cholesterol (HDL-C), triglycerides (TG), fasting blood glucose (FG) and waist circumference (WC), were also examined. Results: Significant reductions (*p* < 0.0001) in SBP/DBP (13 ± 10/7.1 ± 6.6 mmHg) were observed and similarly in pre-diabetic and diabetic patients. Improvements in SBP/DPB were independent of age and gender but greater for elevated baseline SBP/DBP. Tensiofytol^®^ supplementation also significantly improved markers of MetS, with a decrease of TG (11%), WC (1.4%) and FG (4.8%) and an increase of HDL-C (5.3%). Minor side effects were reported in 3.2% patients. Conclusions: This real-life, observational, non-controlled, non-randomized pilot study shows that supplementation of a combination of olive leaf and fruit extracts may be used efficiently and safely in reducing hypertension and MetS markers.

## 1. Introduction

Hypertension is a major component of the metabolic syndrome (MetS), a common cluster of five pre-morbid metabolic-vascular risk factors or disorders associated with increased cardiovascular morbidity, fatty liver disease and risk of cancer. People with MetS meet three or more of the following criteria—enlarged waist circumference (WC) > 88 cm (women) or > 102 cm (men); raised fasting triglycerides (TG) ≥ 150 mg/dL; low high-density lipoprotein cholesterol (HDL-C) < 40 mg/dL (men) or < 50 mg/dL (women); fasting blood glucose (FG) ≥ 100 mg/dL; and/or raised systolic blood pressure (SBP)/diastolic blood pressure (DBP) ≥ 130/≥ 85 mmHg (or ongoing drug treatment for hypertension) [1]. Moreover, increased oxidative stress (OS) has also been accepted in MetS development as a major mechanism for mitochondrial dysfunction, accumulation of protein and lipid oxidation product and impairment of antioxidant systems [2,3].

It has been shown that a 2 mmHg SBP decrease allows reducing the risk of coronary heart disease by 7% and stroke mortality by 10% [4]. Lowering of high blood pressure (BP) can be achieved by antihypertensive drugs such as diuretics, beta-blockers, angiotensin-converting enzyme inhibitors, angiotensin II receptor antagonists or calcium antagonists but also by lifestyle and dietary changes [5]. Thus, the adherence to the traditional Mediterranean-style diet (MedDiet) has been correlated with BP reduction [6,7]. Moreover, a cumulative meta-analysis of prospective studies evidenced a strong inverse association between closer adherence to the MedDiet and the incidence of hard clinical events of cardiovascular vascular diseases (CVD) [8], of collective cardiodiabesity risk (relationship between type 2 diabetes mellitus, obesity, the metabolic syndrome and CVD) [9] and of glycemia [6]. Beneficial effect of MedDiet was attributed to the presence in high amount of polyphenols (phenolic acids and flavonoids) in fruit and vegetables, spices, red wine at moderate intake and olive oil which are typical foods found in the Mediterranean diet [10,11]. The PREDIMED (Prevención con Dieta Mediterránea) study has highlighted the key role of extra-virgin olive oil (EVOO) intake in reducing cardiovascular disease and mortality in individuals at high cardiovascular risk [12], mainly by beneficial effects on BP [6,13,14,15,16,17] and lipids [18] but also by decreasing oxidized low-density lipoprotein (LDL) [19] and inflammation markers [20] and by improving endothelial function [20,21]. The positive effects of EVOO on cardiovascular risk were mainly due to its unique polyphenol content constituted of tyrosol, hydroxytyrosol and oleuropein [22,23], the latter being the most abundant phenolic component in olive tree leaves and olives pulp [24]. Of interest in the use of medicinal plants, olive leaf extracts contain oleuropein, tyrosol and hydroxytyrosol which have notably been linked to some protection against cardiovascular and metabolic diseases [25,26]. Both molecules were also involved in reducing SBP and DBP, low-density lipoprotein cholesterol (LDL-C), inflammatory markers and different parameters of the MetS (fasting blood glucose, low HDL-C or high TG levels) [27,28,29,30,31].

In light of these observations, the goal of the present observational study was to evaluate the supplementation effect and safety of Tensiofytol^®^, a commercially-available combination of *Olea europea* L. leaf and fruit extracts with standardized contents of oleuropein (50 mg/capsule) and hydroxytyrosol (10 mg/capsule), on BP and MetS markers in (pre)-hypertensive patients receiving usual care in real-life practice.

## 2. Material and Methods

### 2.1. Study Design

This observational, non-controlled, non-randomized pilot study was conducted in general practice in Belgium between June 2017 and October 2018. A representative sample of 145 general practitioners (GPs) was randomly selected to incorporate patients suffering from hypertension. They enrolled a total of 663 consecutive patients of their practice (mean 5 ± 2 patients/GP) who basically met the following inclusion criteria—age between 18 and 80 years, suffering from borderline hypertension (SBP of 130 to 139 mmHg and/or DBP of 85 to 89 mmHg) or from Grade 1 hypertension (SBP range of 140–159 mmHg and/or DBP 90–99 mmHg), untreated or under treatment with any BP-lowering medication(s). Pregnant or breast-feeding women were excluded. A questionnaire was completed by the GP for each patient at the first examination (visit 1) before supplementation was initiated. Then patients were instructed to take 2 Tensiofytol^®^ capsules daily for eight weeks with a large glass of water, the first capsule in the morning upon arising and the second before the evening meal. The dosage was adaptable according to the prescriber’s judgment but in this case the dose adjustment had to be stipulated in the survey form. The patients were reviewed by the same GP after two months (visit 2) and a second questionnaire was filled out.

### 2.2. Data Collection

At the initial visit, age, gender, history of antihypertensive medications as well as concomitant treatments with CV and/or glucose-lowering drug(s) were collected for each patient. BP measurements recorded at both visits were performed by general practitioners at the time of the visit, whether morning or afternoon, in their usual way without necessarily using an automated device or referring to conditions recommended in clinical research. From SBP and DBP values, the stage of blood pressure was determined as follows—(1) Optimal BP: SBP < 120 mmHg and DBP < 80 mmHg; (2) Normal BP: SBP of 120–129 mmHg and/or DBP of 80–84 mmHg; (3) Borderline BP (high normal tension): SBP of 130–139 mmHg and/or DBP of 85–89 mmHg; (4) Grade 1 hypertension: SBP of 140-159 mmHg and/or DBP of 90–99 mmHg; (5) Grade 2 hypertension: SBP of 160–179 mmHg and/or DBP of 100–109 mmHg; (6) Grade 3 hypertension: SBP ≥ 180 mmHg and/or DBP ≥ 110 mmHg and (7) Isolated systolic hypertension: SBP ≥ 140 mmHg and DBP < 90 mmHg. Markers of the MetS, consisting of WC (cm), TG (mg/dL), HDL-C (mg/dL) and FG (mg/dL) levels were also collected at both visits but on an optional basis. At visit 2 (after 2 months), the patient was questioned about the occurrence/description of side effects and about his/her intention to continue the supplementation with Tensiofytol^®^. Dates of visits were carefully noted and a 10-day window (50–70 days) was normally allowed between visits.

According to the Belgian law of 7 May 2004 (Art. 3 §2, http://www.ejustice.just.fgov.be/cgi_loi/change_lg.pl?language=fr&la=F&cn=2004050732&table_name=loi) on human experiments, seeking approval by an ethical committee for a post-hoc observational study of data collected from the records of general practitioners (GPs) is not required. Moreover, the dietary supplement taken in accordance with good medical practice, without patient’s assignment to a given therapeutic strategy, as the decision to take Tensiofytol, already on the market and available without prescription in Belgium, was not related to the study and did not require additional diagnostic or monitoring procedures.

### 2.3. Intervention

Tensiofytol^®^ (Tilman SA, Baillonville, Belgium) is an olive tree leaf and olive fruit dry extracts-based food supplement which contributes to the maintenance of normal BP. As shown in Table 1, each Tensiofytol^®^ capsule contains 167 mg of standardized *Olea europea* leaf dry extract (ethanol-water mixture as extraction solvent, equivalent to 50 mg of oleuropein) and 53 mg of standardized *Olea europea* L. fruit dry extract (water as extraction solvent, equivalent to 10 mg of hydroxytyrosol). The posology recommended in the study (namely a daily intake of 2 Tensiofytol^®^ capsules) provides 100 mg/d of oleuropein and 20 mg/d of hydroxytyrosol.

### 2.4. Statistical Analyses

For quantitative variables, results were expressed as mean and standard deviation (SD), whereas frequency tables (number and percent) were used for categorical variables. The statistical analysis was done on data from the “intention-to-treat” population consisting of all study participants without restrictions. Missing data were not replaced nor imputed. To assess supplementation effect, parameter changes between the two visits were calculated and tested by the paired Student *t*-test. Supplementation effect was adjusted for baseline factors by multiple regression analysis where factor effects were displayed as regression coefficients and their standard error (SE). The statistical analysis was also done separately on pre-diabetic (FG ≥ 100 mg/dL but < 126 mg/dL) and diabetic patients (FG ≥ 126 mg/dL). All tests were two-sided and considered significant at 5% critical level (*p* < 0.05). Statistical analyses were performed using SAS software (Version 9.4) and graphics with R software (version 3.2.5).

## 3. Results

### 3.1. Patient Characteristics

Patient characteristics at baseline visit are displayed in Table 2. The mean age was 60 ± 12 years (range: 18–95 years) and the proportion of female patients was 50.7%. Globally, 45.7% patients had a history of hypertensive treatment. The mean SBP and DBP were 151 ± 11 mmHg and 91 ± 6.6 mm Hg, respectively. Based on these measurements, 48.7% of patients suffered from Grade 1 hypertension and 5.1% from borderline tension. MetS markers were on average 148 ± 66 mg/dL for TG (*n* = 416), 101 ± 19 mg/dL for FG (*n* = 426), 55 ± 17 mg/dL for HDL-C (*n* = 404) and 98 ± 14 cm for WC (*n* = 270), respectively.

### 3.2. Supplementation Initiation

Tensiofytol^®^ was initiated according to the recommended posology—1 capsule twice daily before meals, in 640 patients (96.5%). The dosage was adjusted for 11 subjects [once a day (*n* = 9), 2 capsules in the morning (*n* = 1) or 2 capsules twice a day (*n* = 1)]. The adaptation of the dosage regimen was not specified for 12 patients. All patients were reviewed at a second visit after a mean follow-up of 68 ± 19 days. A total of 399 (60.2%) patients took Tensiofytol^®^ capsules as sole supplementation. For the remaining participants, the different types of medications taken simultaneously for elevated BP or other conditions are presented in Table 2.

### 3.3. Supplementation Effect

#### 3.3.1. Effects on Blood Pressures

As seen in Table 3, mean SBP and DBP were significantly reduced from baseline after a 2-month intake of Tensiofytol^®^ capsules (*p* < 0.0001). The mean lowering effect observed was 13 ± 10 mmHg and 7.1 ± 6.6 mmHg for SBP and DBP, respectively. Multiple regression analysis applied to SBP and DBP differences showed that supplementation effect was not influenced by gender, history of antihypertensive treatment or other concomitant drugs, only a borderline effect being observed for age on SBP. By contrast, the decrease of both BP levels was significantly higher for patients with elevated BP values (*p* < 0.0001 for SBP and DBP) at baseline (Table 4).

#### 3.3.2. Effect on Markers of Metabolic Syndrome

In patients with MetS parameters available at both visits, the 2-month intake of Tensiofytol^®^ also evidenced significant improvements (Table 3). Specifically, subjects exhibited a significant mean reduction of 18 ± 42 mg/dL in TG levels (*p* < 0.0001, *n* = 162), of 4.9 ± 11 mg/dL in FG levels (*p* < 0.0001, *n* = 186) and of 1.4 ± 3.2 cm in WC (*p* < 0.0001, *n* = 170). By contrast, HDL-C levels significantly increased by 2.8 ± 8.1 mg/dL (*p* < 0.0001, *n* = 160). In terms of baseline levels, Tensiofytol^®^ supplementation improved markers of MetS by 11% for TG, 4.8% for FG, 5.3% for HDL-C and 1.4% for WC.

#### 3.3.3. Side Effects

Side effects were reported by 21 (3.2%) patients, including headaches (*n* = 2), gastro-intestinal complaints such as nausea, diarrhea and cramps (*n* = 9), vertigo (*n* = 1), itchy leg (*n* = 1) and unspecified adverse events (*n* = 8). A vast majority of patients (88.8%) wished to continue taking the dietary supplement beyond the scheduled completion of the study; 10.7% opted for discontinuation of the supplementation after study completion and data were not available for 0.45% of patients.

#### 3.3.4. Pre-Diabetic and Diabetic Patients

Based on FG measurements available at baseline (*n* = 426), 134 (31.5%) patients were pre-diabetic (FG > 100 mg/dL but ≤ 126 mg/dL) and 44 (10.3%) diabetic (FG > 126 mg/dL). Their characteristics are given in Table 2. Borderline tension was present in 10 pre-diabetic subjects (7.5%) and 2 diabetic patients (4.5%), whereas Grade 1 hypertension affected 68 pre-diabetic participants (50.8%) and 27 diabetic patients (61.4%). Over 50% had been previously treated with antihypertensive medications in both groups. Table 3 shows that Tensiofytol^®^ supplementation was also associated with a significant decrease of BP in pre-diabetic and diabetic patients (*p* < 0.0001). Mean SBP lowered by 11 ± 9.2 mmHg in pre-diabetic patients and by 13 ± 9.4 mmHg in diabetics. For DBP, reductions averaged 6.8 ± 6.1 mmHg and 7.7 ± 8.0 mmHg, respectively. As for MetS markers, the largest effects were observed among diabetic subjects with an HDL-C increase of 6.7 ± 11 mg/dL (*p* = 0.018, *n* = 18) and a mean drop of 59 ± 70 mg/dL (*p* = 0.0016, *n* = 19) for TG, of 19 ± 16 mg/dL (*p* < 0.0001, *n* = 24) for FG and of 2.0 ± 2.5 cm (*p* = 0.0012, *n* = 22) for WC.

## 4. Discussion

The present pilot study conducted in a real-life general practice setting focused on the supplementation effect and safety of Tensiofytol^®^, a combination of olive leaf and fruit dry-extracts, in the clinical management of hypertension and metabolic syndrome.

### 4.1. Effect on Blood Pressures

According to the recent ESC/ESH guidelines for the management of arterial hypertension [5], a 10 mmHg reduction in SBP or a 5 mmHg reduction in DBP was associated with significant reductions in all major CV events by circa 20%, all-cause mortality by 10–15%, stroke by 35%, coronary events by 20% and heart failure by 40%. These relative risk reductions are consistent, irrespective of baseline BP within the hypertensive range, the level of CV risk, comorbidities (e.g., diabetes and chronic kidney disease), age, gender and ethnicity. Our study demonstrated that the daily intake during 8-weeks of olive leaf and fruit dry extracts-based supplement (Tensiofytol^®^) at recommended posology (100 mg oleuropein and 20 mg hydroxytyrosol/day) was associated with significant reductions from baseline in mean SBP and DBP of 13 ± 10 mmHg and 7.1 ± 6.6 mmHg, respectively. Patients with elevated baseline BP experienced greater BP-lowering. History of antihypertensive treatment or other concomitant treatments had no impact on these findings. Additional subgroups analyses performed on pre-diabetic and diabetic participants led similar findings. As seen in Table 5, our data confirmed the beneficial effects of different *Olea europea* L. leaf extracts on hypertension found in other studies [27,28,29,30,31] but with a much larger sample size (*n* = 663). By comparison with Tensiofytol^®^, Susalit et al. [29] demonstrated that captopril as antihypertensive drug administered daily at a dose 25–50 mg in stage-1 hypertensive patients was able to reduce in a similar manner mean BP from baseline to the end of study (13.7 ± 7.6 mmHg and 6.4 ± 5.2 mmHg for SBP and DBP, respectively). The anti-hypertensive effect of Tensiofytol^®^ demonstrated here is in line with other studies concluding that consumption of a diet containing polyphenol-rich olive oil can decrease BP and protect the cardiovascular system by improving endothelial function and enhancing the endothelial synthesis of NO known for its potent vasodilating properties [16,17,19,32,33]. Using Endo-Pat 2000 device (Itamar Medical, Israel) which records endothelium-mediated changes in the digital pulse waveform measured with a pair of novel modified plethysmographic probes situated on the finger index of each hand [34], we have shown that Tensiofytol^®^ caused 1 h after intake of 2 capsules a significant improvement (20%) of endothelial function (measured as reactive hyperemia index (RHI) defined as transient increase in organ blood flow that occurs following a brief period of arterial occlusion). Such observations have also been reported after intake of olive oil when compared to basal value. However, this increase was more marked (41%) in patients having a low basal RHI value [34].

### 4.2. Effects on MetS Markers

The study also showed that 2-month supplementation with Tensiofytol^®^ improved markers of MetS by significantly reducing mean levels of TG, FG and WC, while increasing HDL-C level. Analyses performed on pre-diabetic and diabetic participants showed significant beneficial effects on markers of MetS for these patients, with remarkable results obtained for the diabetic patient subgroup, with an increase of 6.7 mg/dL for HDL-C and significant decreases of TG (59 mg/dL), FG (19 mg/dL) and WC (2.0 cm). The above-cited studies focusing on the antihypertensive effects of olive leaf extracts also highlighted some positive effects on LDL-C and markers of the MetS after supplementation with various olive leaf extracts. Perrinjaquet-Moccetti et al. [28] reported a significant dose-dependent decrease in LDL-C level for both olive extract groups vs placebo, while decreases in TG, total cholesterol and LDL-C level were observed for the dietary supplementation group (200 mg oleuropein/d) vs. captopril group in another study with the same extract [29]. Similarly, reductions in plasma total cholesterol, LDL-C and TG as well as interleukin-8 levels were also noticed with other olive leaf extracts [30,31] and a light decrease of glycemia was observed in one study [27]. Compared to these studies, the daily dosage of oleuropein was equal or lower with Tensiofytol^®^ capsules, showing nevertheless promising or better results on BP and MetS parameters. Tensiofytol^®^ capsules formulation also includes a fruit extract from *Olea europea* L., corresponding to 20 mg of hydroxytyrosol a day at the prescribed posology. This fruit extract, combined to red yeast rice, already showed a significant lowering effect on SBP and DBP of 10 mmHg and 7 mmHg, respectively, while significant drops were seen for total cholesterol (17%), LDL-C (24%), oxidized LDL-C (20%) and TG (9%) [35,36]. MetS markers were also positively impacted by high olive-derived polyphenol-content diet. It was shown that polyphenol-enriched olive oil improves HDLs characteristics in hypercholesterolemic subjects [37] while the Framingham study suggested that a 1 mg/dL increase in HDL-C was associated with a 2% decrease (in men) and a 3% decrease (in women) in risk of coronary heart disease [38]. Promising effects of olive polyphenols were also observed on glycemia and diabetes [39] and it was shown that supplementation with an olive leaf extract was associated with significant lowering of HbA1c and fasting plasma insulin levels [40]. Another study also observed that supplementation with 51.1 mg oleuropein and 9.7 mg hydroxytyrosol per day for 12 weeks was associated with a 15% improvement in insulin sensitivity compared to placebo and that there was also a 28% improvement in pancreatic B-cell responsiveness [41].

### 4.3. Potential Underlying Mechanisms of Action

Oxidative stress (OS) has been defined as an imbalance between reactive oxygen species (ROS) and antioxidants in favor of oxidants, leading to a disruption of redox signaling and/or irreversible oxidative damage to lipids, DNA or proteins [42]. It has been recognized to play a key role major role in the pathophysiology of hypertension-induced endothelial dysfunction and in the development of MetS. Due to its potential ability to scavenge ROS as evidenced in in vitro and animal studies, hydroxytyrosol and oleuropein have been proposed to exert positive modulation effects in cardiovascular system and metabolic syndrome [43]. In a randomized double-blinded, placebo-controlled crossover trial on 28 healthy volunteers (age: 18 to 65 years), Colica et al. [44] showed that an intake of 15 mg/day of pure hydroxytyrosol for a 3-week period resulted in a strengthening of the antioxidant capacity of the plasma (thiol proteins, superoxide dismutase, total antioxidant status) and also a decrease in plasma malondialdehyde (MDA) concentration. Garcia-Villalba et al. [45] reported that an intake of 250 mg olive leaf extract contributed to an increase of the total plasma antioxidant status. However, these results need to be taken with caution because the detection of MDA by the thiobarbituric assay (TBARS) was not specific evidence of a lipid peroxidation process. Moreover, it was not established that changes in the overall antioxidant capacity of plasma exerts a beneficial physiological effect in humans as required by Regulation (EC) 208 No 1924/2006. More interestingly, hydroxytyrosol, but also oleuropein, have been reported to inhibit, in a dose-dependent way, LDL and HDL oxidation both in in vitro and in vivo, by repressing ROS-driven reactions [46,47].

After intake of 250 mg olive leaf extract, Garcia-Villalba et al. [45] identified three metabolites derived from hydroxytyrosol (sulfoglucuronide, glucuronide, sulfate), four oleuropein aglycone derivatives and two homovanillic acid metabolites in plasma and urine, in agreement with studies using olive oil [48]. Miro-Casas et al. [49] reported that 98% of hydroxytyrosol can be detected in both plasma and urine in conjugated forms, mainly glucuronoconjugates and only 2% as its free form within 0–12 h after intake of 25 mL virgin olive oil. Plasma concentration in hydroxytyrosol was 25.83 µg/L (0.16 µM) two hours after intake. Pastor et al. [50] reported that free hydroxytyrosol plasma concentrations following the intake of 25 mL of an extra-virgin olive oil (4.40 ng/mL) was 4.5 µg/L (0.025 µM) half an hour after ingestion. By contrast, Colica et al. [44] reported that the intake of pure hydroxytyrosol (15 mg/day for 3 weeks) led to a plasma concentration equal to 2.83 mg/L (18 µM). By comparison, blood concentration in three major antioxidants such as vitamin C, vitamin E and glutathione were respectively of 60, 30 and 600 µM. Thus, the beneficial health effect of hydroxytyrosol but also of polyphenols in general could not be attributed in any way to a direct ROS scavenging activity due to their very low plasma concentrations (around or less than 1–10 µM). Whether plasma hydroxytyrosol per se or its metabolites and this true for all phenolic compounds, were responsible of the beneficial health effect remains an open question [51]. As an example, Pourova et al. [52] reported that quercetin metabolites (3,4-dihydroxyphenylacetic and 4-methylcatechol) could also have, in addition to quercetin itself, the capacity to decrease blood pressure by inducing a vasorelaxation effect in endothelial cells. In an interventional study of 11 healthy volunteers whose diet was supplemented with 50 mL of virgin olive oil, Khymenets et al. [53] showed, however, that the main metabolites of hydroxytyrosol (3′-*O*- and 4′-*O*-glucuronides) at concentrations found in urine (0.01–10 µM) were unable to display any significant antioxidant activities.

In order to explain the potential antioxidant effect of hydrotyrosol, recent studies rather highlighted the modulation of cellular pathways by olive oil phenolic compounds through a moderate pro–oxidant effect (hormesis) resulting from the hydroxyl groups auto-oxidation inside the cells [54]. In response to ROS production slightly higher than their physiological concentration, cells stimulated the expression of intracellular antioxidant enzymes (e.g., heme oxygenase or HO-1) through activation of Nuclear factor erythroid 2-related factor 2 (NrF2) transcription [55]. Olive oil, hydroxytyrosol, tyrosol and oleuropein were all able to stimulate the NrF2 pathway [56,57,58]. Of interest, a novel potential therapeutic approach in metabolic syndrome and obesity proposed the use of hormetic molecules to induce NrF2-mediatedcytoprotection against oxidative stress and inflammation [59,60]. Independently, the components of metabolic syndrome each have the potential to affect the endothelium resulting in reduced nitric oxide production, increased reactive oxygen species and increased production of vasoconstrictors [61]. It is well known that endothelial dysfunction increases risk of developing atherosclerosis, insulin resistance, metabolic syndrome and diabetes mellitus type 2 [62]. In both cells and animal models, hydroxytyrol and oleuropein have been shown able to regulate NO production and modulate upwards the activity of endothelial nitric oxide synthase (eNOS) [63,64] even if Schmit et al. in their paper [65] reported no effect of hydrotyrosol on human endothelial cells. MetS was also associated to low-grade inflammation characterized by abnormal pro-inflammatory cytokine, activation of inflammatory signaling pathways and increased ROS production in adipose tissues [66]. Many studies have reported that inflammation and oxidative stress pathophysiological processes were closely related, one being easily induced by the other [67,68]. There is now extensive experimental and clinical evidence indicating that hypertension is associated with inflammation driven in large part by oxidative stress [69,70]. In both cellular and animal models, hydroxytyrosol has been shown to exhibit anti-inflammatory properties [71,72].

### 4.4. Safety and Side Effects

Overall, the supplementation was well tolerated with a marginal rate of non-serious side effects (3%, mainly headaches, nausea, diarrhea or cramps) and a high rate of intended persistence of the supplementation (89%).

### 4.5. Strength and Limitations

The observational, non-placebo-controlled nature of the study is a limitation and findings will have to be confirmed by randomized controlled trials. The conduct of RCTs in general practice is unusual and by far more difficult than in clinical settings. Therefore, the recourse to observational studies as this one involving hundreds of patients is still of interest in assessing the potential effect of supplementation with herbal products in real-life medical practice [73,74,75]. The absence of randomization and control cannot preclude the positive role of potential confounders, including older age, natural compound instead of synthetic drug, changes in lifestyle habits over the study period in patients openly treated with a natural product that could be perceived as intrinsically healthy and beneficial. BP was measured only once at each patient’s visit and not necessarily with an automated system and in standard conditions. This has certainly impacted the quality and accuracy of measurements. However, since study findings were mainly based on blood pressure differences between visits, the potential bias in BP measurements may have been partially attenuated. Finally, further investigations should be performed to check the bioavailability of phenolic compounds present in Tensiofytol^®^ capsules.

## 5. Conclusions

Clinically relevant improvements in blood pressure and in MetS markers were obtained in (pre)-hypertensive patients with Tensiofytol^®^ capsules. Based on study findings and other evidence, the use of this olive leaf and fruit dry extracts-based supplementation, in association with a coherent improvement in diet and lifestyle, could represent a non-pharmacological alternative for managing pre-hypertensive patients and an effective adjuvant, together with pharmacological treatment, for hypertensive patients.

## Figures and Tables

**Table 1:** Composition per capsule of Tensiofytol in decreasing order according to Regulation (EU) No 1169/2011 Article 18 and Annex VII.

Name	Part Used/Form	Botanical Name	Category	Numero E	Quantity API	Unit
Olive	Leaf, dry ext.	*Olea europaea* L.			167	mg
Gelatin (Capsule shell)	Bovine					
Olive	Fruit, dry ext.	*Olea europaea* L.			53	mg
Microcrystalline cellulose			Bulking agent	E 460(i)		
Tricalcium phosphate			Bulking agent	E341(iii)		
Talc			Anticaking agent	E 553b		
Silicon dioxide			Anticaking agent	E 551		
Magnesium salts of fatty acids	Magnesium stearate		Anticaking agent	E 470b		
Iron oxide yellow			Color	E 172		
Iron oxide black			Color	E 172		

**Table 2:** Baseline characteristics of patients of the total population, pre-diabetic and diabetic groups.

Variable	All Patients	Pre-Diabetic *	Diabetic **
Number (*n*) of patients	663	134	44
Age (years), mean ± SD	60 ± 12	61 ± 12	62 ± 10
Gender (female), *n* (%)	336 (50.7)	57 (42.5)	19 (43.2)
History of AHT, *n* (%)	303 (45.7)	71 (53.0)	26 (59.1)
SBP (mmHg), mean ± SD	151 ± 11	149 ± 10	150 ± 8.6
DBP (mmHg), mean ± SD	91 ± 6.6	91 ± 6.2	91 ± 5.3
Stage of blood pressure, *n* (%)			
Optimal tension	1 (0.2)	0 (0.0)	0 (0.0)
Normal tension	1 (0.2)	1 (0.7)	0 (0.0)
Borderline tension	34 (5.1)	10 (7.5)	2 (4.5)
Grade 1 hypertension	323 (48.7)	68 (50.8)	27 (61.4)
Grade 2 hypertension	122 (18.4)	28 (20.9)	5 (11.4)
Grade 3 hypertension	18 (2.7)	1 (0.7)	1 (2.3)
Isolated systolic hypertension	164 (24.7)	26 (19.4)	9 (20.4)
Concomitant therapy, *n* (%)			
None - Tensiofytol^®^ alone	399 (60.2)	69 (51.5)	8 (18.2)
Antihypertensive concomitant treatment	92 (13.9)	20 (14.9)	8 (18.2)
*Antihypertensive treatment alone*	*52 (7.8)*	*9 (6.7)*	*0 (0.0)*
*Antihypertensive and CV treatments*	*21 (3.2)*	*4 (3.0)*	*1 (2.3)*
*Antihypertensive and diabetes treatments*	*12 (1.8)*	*2 (1.5)*	*6 (13.6)*
*Antihypertensive, CV and diabetes treatments*	*7 (1.1)*	*5 (3.7)*	*1 (2.3)*
Non-antihypertensive concomitant treatment	172 (25.9)	45 (33.6)	28 (63.6)
*Other therapy alone*	*13 (2.0)*	*2 (1.5)*	*0 (0.0)*
*Other therapy and CV treatment*	*92 (13.9)*	*22 (16.4)*	*1 (2.3)*
*Other therapy and diabetes treatment*	*36 (5.4)*	*11 (8.2)*	*16 (36.3)*
*Other therapy and CV/diabetes treatments*	*31 (4.7)*	*10 (7.5)*	*11 (25.0)*

* Pre-diabetic (Fasting blood glucose > 100 mg/dL but ≤ 126 mg/dL); ** Diabetic (Fasting blood glucose > 126 mg/dL); AHT Previous antihypertensive treatment; SBP Systolic Blood Pressure; DBP Diastolic Blood Pressure; V Cardiovascular.

**Table 3:** Supplementation effect on blood pressure parameters and metabolic syndrome markers in the total study population and in the pre-diabetic and diabetic groups.

Variable	Number of Patients *	Visit 1	Visit 2	Difference	*p*-Value
Total population					
SBP (mmHg)	663	151 ± 11	138 ± 10	−13 ± 10	<0.0001
DBP (mmHg)	663	91 ± 6.6	84 ± 6.8	−7.1 ± 6.6	<0.0001
Triglycerides (mg/dL)	162	159 ± 70	140 ± 54	−18 ± 42	<0.0001
Fasting glucose (mg/dL)	186	102 ± 19	98 ± 15	−4.9 ± 11	<0.0001
HDL-Cholesterol (mg/dL)	160	53 ± 16	55 ± 16	2.8 ± 8.1	<0.0001
Waist circumference (cm)	170	100 ± 16	98 ± 16	−1.4 ± 3.2	<0.0001
Pre-diabetic group					
SBP (mmHg)	134	149 ± 10	138 ± 9.5	−11 ± 9.2	<0.0001
DBP (mmHg)	134	91 ± 6.2	84 ± 6.3	−6.8 ± 6.1	<0.0001
Triglycerides (mg/dL)	57	165 ± 50	147 ± 44	−19 ± 33	<0.0001
Fasting glucose (mg/dL)	67	108 ± 6.8	103 ± 8.7	−5.2 ± 7.8	<0.0001
HDL-Cholesterol (mg/dL)	57	49 ± 12	52 ± 13	2.5 ± 6.3	0.0043
Waist circumference (cm)	67	102 ± 16	100 ± 16	−1.9 ± 3.9	0.0001
Diabetic group					
SBP (mmHg)	44	150 ± 8.6	137 ± 9.8	−13 ± 9.4	<0.0001
DBP (mmHg)	44	91 ± 5.3	84 ± 7.3	−7.7 ± 8.0	<0.0001
Triglycerides (mg/dL)	19	236 ± 105	176 ± 90	−59 ± 70	0.0016
Fasting glucose (mg/dL)	24	141 ± 14	122 ± 16	−19 ± 16	<0.0001
HDL-Cholesterol (mg/dL)	18	40 ± 10	47 ± 9.6	6.7 ± 11	0.018
Waist circumference (cm)	22	104 ± 18	102 ± 18	−2.0 ± 2.5	0.0012

Results are expressed as Mean ± SD; * Patients with data available at both visits; DBP Diastolic Blood Pressure; SBP Systolic Blood Pressure; HDL-C High-Density Lipoprotein Cholesterol.

**Table 4:** Baseline factors associated with systolic blood pressure (SBP) and diastolic blood pressure (DBP) changes after 2-month supplementation with Tensiofytol^®^.

Factor	Coefficient ± SE *	*p*-Value
SBP change (mmHg)		
Age (years)	0.060 ± 0.029	0.044
Gender (Female)	−0.63 ± 0.68	0.36
Baseline SBP (mmHg)	−0.51 ± 0.032	<0.0001
History of antihypertensive drugs (yes)	−0.40 ± 0.72	0.58
Other concomitant therapy (yes)	−0.75 ± 0.74	0.31
DBP change (mmHg)		
Age (years)	0.0078 ± 0.020	0.69
Gender (Female)	−0.71 ± 0.46	0.12
Baseline DBP (mmHg)	−0.48 ± 0.035	<0.0001
History of antihypertensive drugs (yes)	−0.50 ± 0.49	0.31
Other concomitant therapy (yes)	−0.65 ± 0.50	0.19

* Derived by multiple regression analysis; a positive (negative) coefficient is associated with a lower (higher) reduction in blood pressure after treatment; SBP Systolic Blood Pressure: DBP Diastolic Blood Pressure.

**Table 5:** Comparison of studies on blood pressure reduction after treatment with oleuropein and hydroxytyrosol.

Study	*n*	Oleuropein and Hydroxytyrosol Content (mg/d)	Treatment Duration (weeks)	Baseline SBP (mmHg)	SBP Reduction (mmHg)	Baseline DBP (mmHg)	DBP Reduction (mmHg)
Cherif et al., [27]	30	115 and NA *	12	164.5 ± 20.9	16.7 (*p* < 0.001)	97.4 ± 9.8	10.4 (*p* < 0.001)
Perrinjaquet-Moccetti et al., [28]	10	208 and NA	8	137 ± 10	11.0 (*p* < 0.01)	80 ± 10	4.0 (NS)
Susalit et al., [29]	72	200 and NA	8	145.0 ± 5.0	11.5 ± 8.5 (*p* < 0.001)	91.3 ± 5.1	4.8 ± 5.5 (*p* < 0.001)
Cabrera-Vique et al., [30]	10	240 and 16	4	133.2 ± 3.4	11.2 (*p* = 0.011)	80.9 ± 2.1	8.0 (*p* = 0.026)
Lockyer et al., [31]	60	136 and 6	6	139.69 ± 12.28	3.95 ± 11.48 (*p* = 0.027)	83.71 ± 8.86	3.00 ± 8.54 (*p* = 0.025)
Hermans et al., [present data]	663	100 and 20	8	151 ± 11	13 ± 10 (*p* < 0.0001)	91 ± 6.6	7.1 ± 6.6 (*p* < 0.001)

* NA not available.
